# An IoT-Based Road Bridge Health Monitoring and Warning System

**DOI:** 10.3390/s24020469

**Published:** 2024-01-12

**Authors:** A. R. Al-Ali, Salwa Beheiry, Ahmad Alnabulsi, Shahed Obaid, Noor Mansoor, Nada Odeh, Alaaeldin Mostafa

**Affiliations:** 1 Department of Computer Science and Engineering, American University of Sharjah, Sharjah P.O. Box 26666, United Arab Emirates; aalnabulsi@aus.edu (A.A.); g00084579@aus.edu (S.O.); g00083854@alumni.aus.edu (N.M.); g00083534@alumni.aus.edu (N.O.); b00077452@alumni.aus.edu (A.M.); 2 Department of Civil Engineering, American University of Sharjah, Sharjah P.O. Box 26666, United Arab Emirates; sbeheiry@aus.edu

**Keywords:** resilient infrastructure, fuzzy logic, intelligent transport systems, internet of things, smart road bridges, structural health monitoring systems, UNSDGs

## Abstract

Recent earthquakes worldwide have led to significant loss of life and structural damage to infrastructure, especially road bridges. Existing bridge monitoring systems have limitations, including restricted detection capabilities, subjectivity, human error, labor-intensive inspections, limited access to remote areas, and high costs. Aging infrastructures pose a critical concern for organizations and government funding policies, showing signs of decay and impending structural failure. To address these challenges, this research proposes an IoT-based bridge health status monitoring and warning system that is wireless, low-cost, durable, and user-friendly. The proposed system builds upon engineering standards and guidelines to classify bridge health status into categories ranging from excellent to collapse condition. It incorporates deflection, vibration, temperature, humidity, and infrared sensors, combined with IoT and a fuzzy logic algorithm. The primary objective is to reduce bridge maintenance costs, extend lifespans, and enhance transportation safety through an early warning system via a mobile application. Additionally, a Google Maps interface has been developed to display bridge conditions along with real-time traffic video. To validate the proposed system, a 3-D prototype model was constructed and tested. Practical testing of the fuzzy logic algorithm aligned with the simulation outcomes, demonstrating expected accuracy in determining bridge health status.

## 1. Introduction

Road bridge failures can have severe ramifications on transportation systems worldwide. Apart from the loss of lives and casualties, the disruption in services leads to highly adverse effects on economic growth. Throughout history, numerous bridge failures have resulted in tragic consequences, including human fatalities and substantial economic losses. A recent example is the collapse of Italy’s Morandi’s Polcevera Viaduct in 2018, resulting in 43 deaths and nine injuries as well as an estimated economic loss of approximately 100 million yuan (approximately 14 million dollars) [1].

In the United States, according to the latest Report Card for America’s Infrastructure, 42% of the 617,000 country’s bridges are at least 50 years old; among these, 7.5% fall under the classification of “structurally deficient” [2]. 

China, a country with massive bridge production, had a total number of 851,500 bridges by the end of 2018 [3]. Despite this extensive infrastructure, the country has encountered several serious bridge collapses [4]. Road bridge failures are attributed to a range of factors, including natural disasters, aging infrastructure, environmental conditions, deficiencies in structural design, material degradation due to environmental conditions, increased traffic loads, insufficient maintenance and inspection, and human error [1,5,6]. 

Developing nations often face a myriad of challenges when it comes to bridge infrastructure development and monitoring. Some of the specific challenges include financial constraints; geopolitical and environmental factors; technology limitations; maintenance and upkeep; access to skilled labor; corruption and governance issues; inadequate planning; and, above all, impact on local sustained communities. Addressing these challenges requires a holistic approach involving technology development by academia and industry, government commitment, international support, improved governance, technology transfer, capacity building, and sustainable financing strategies. Collaboration among governments, private sectors, and international organizations is crucial to overcoming these obstacles and ensuring the successful development of bridge infrastructure in developing nations [7,8,9,10].

In response to these challenges, the integration of advanced technologies, such as the Internet of Things (IoT) and Artificial Intelligence (AI), into infrastructure development and monitoring systems emerges as a promising solution. Emphasizing energy efficiency and resource optimization, IoT and AI technologies can actively enhance public health and contribute to fostering a circular economy [11]. This commitment to sustainable practices, taking into consideration the environmental factors, aligns seamlessly with the United Nations Sustainable Development Goals (UNSDGs) [11,12,13,14,15,16,17,18,19,20,21,22]. 

Through the utilization of sensors and smart devices, these advanced technologies play a crucial role in obtaining, transmitting, retaining, and analyzing real-time data. This not only offers significant insights into the safety and state of bridges but also provides valuable information for guaranteeing structural soundness, optimizing maintenance strategies, and enhancing the overall safety of transportation infrastructure [12].

Within the framework of sustainable community development, the utilization of IoT and AI technologies in bridge monitoring systems goes beyond infrastructure concerns. It facilitates the monitoring of earth systems and aids in adapting to climate change and other global-scale issues. This is realized by providing valuable data for decision support systems and actively contributing to the development of sustainable energy systems and infrastructures [13].

Moreover, these technologies seamlessly integrate circular economy principles into infrastructure development, aiming to minimize waste, maximize resource efficiency, and promote sustainability. This involves prioritizing longevity, durability, improved material selection, integrating renewable energy techniques, and implementing smart technologies for monitoring, maintenance, and resource utilization efficiency [15,16,17]. By incorporating circular economy principles, nations can reduce resource consumption, minimize waste, lower environmental impacts, and establish more sustainable and resilient systems for current and future generations. Resilient infrastructure is strategically designed and planned to survive the impact of wear and tear and natural disasters like floods, earthquakes, or wildfires. Most importantly, resilience is the concept of bouncing back from failure in a timely and reliable manner. 

An integral aspect of resilient systems lies in their consideration of environmental factors, a thoughtful approach that is essential for creating sustainable and environmentally friendly systems that benefit society, protect ecosystems, and contribute to a healthier planet for future generations [18,19,20]. This involves reducing resource consumption, mitigating pollution, and preserving natural habitats. Infrastructure projects often include land use changes, construction, and alterations to waterways, and such considerations help minimize disruption to ecosystems, preserving biodiversity and protecting wildlife habitats. Furthermore, infrastructure designed with careful consideration for the environment demonstrates enhanced resilience against the impacts of climate change, including extreme weather events, rising sea levels, and shifting precipitation patterns. Environmental considerations in infrastructure design promote the efficient use of resources such as energy, water, and materials while employing green technologies, renewable energy sources, and sustainable materials to reduce resource consumption and waste generation. Careful planning and design minimize negative environmental impacts, including measures to control air and water pollution, reduce noise levels, and manage construction waste, ultimately contributing to a reduction in the carbon footprint.

It is clear from the reviewed literature that a gap exists in terms of affordable systems that can be applied in developing countries. This gap is particularly relevant to the achievement of the UN Sustainable Development Goals (UNSDG) #9—building resilient infrastructure to promote inclusive and sustainable industrialization and foster innovation, as well as goal #11—make cities and human settlements inclusive, safe, resilient, and sustainable [23]. To address this gap, this paper proposes an IoT-Based Smart Road Bridge Health Monitoring and Warning System. The system’s objective is to enhance existing solutions by providing a low-cost autonomous bridge health monitoring system to address a spectrum of issues associated with bridge safety and management. By using a set of sensors that are embedded across multiple nodes of the bridge at hand (Data Acquisition System), the system aims to monitor and analyze the conditions of the bridge, including bridge stiffness and response to load, vibration levels, traffic count, and other safety conditions. The sensor readings are to be processed by an edge computing device such as a Raspberry Pi to analyze the status of the bridge.

The proposed system uses fuzzy logic algorithms to analyze the monitored bridge health parameters according to a set of rules that are based on the membership functions, then categorize the bridge health status into a specific level (Excellent, Safe, Fair, Critical, and Collapse) [24]. Moreover, these key parameters and the bridge health status will be transmitted to Firebase, a cloud-hosted database that synchronizes data in real-time to every connected client. We have developed a dashboard that shows the bridge status from anywhere, at any time. In addition, a mobile application will be developed to allow relevant technicians and engineers access to reports and alerts of the bridge’s health status. 

The proposed system integrates the advantages of both sensor-based and vision-based systems, offering a robust solution for bridge health monitoring. With its diverse range of sensors, the system can deliver accurate and quantitative data on the bridge’s behavior, facilitating precise analysis and assessment of its structural health. These sensors are adaptable to various environmental conditions, ensuring reliable and continuous monitoring. Furthermore, the integration of cameras in the system enables efficient traffic counting and real-time visual monitoring, enhancing the system’s capabilities and providing valuable insights for bridge management and safety.

The upcoming sections of this paper are organized as follows: a comprehensive review of the existing literature is provided in Section 2. Section 3 shows the system requirements, followed by the hardware architecture in Section 4. The system software algorithm will be presented in Section 5. Testing, validation, and discussion are presented in Section 6. Finally, Section 7 concludes the paper and provides possible future direction.

## 2. Literature Review

Numerous scholarly works have explored Road Bridge Health Monitoring and Warning Systems, with a primary focus on investigating the impacts of factors such as stiffness loss, time- and temperature-dependent deformations, fatigue, corrosion, scour, and vibration when assessing the health status of a bridge. To reliably detect such damages, continuous monitoring using state-of-the-art methods and techniqvues is essential [1,4,5,6]. The rise of IoT and AI applications has led to the implementation of smart bridge health monitoring systems in several countries. For instance, the Governor Mario M. Cuomo Bridge in New York features one of the most complex and advanced monitoring systems for bridge health [25].

This review organizes these scholarly efforts based on the technologies employed and the parameters under consideration. These systems can be broadly categorized into two main groups:

- Sensor-Based Systems: These systems utilize a variety of sensors, such as deflection sensors, vibration sensors, strain sensors, temperature sensors, and humidity sensors to continuously monitor the structural health of the bridge and detect any potential issues.
- Vision-Based Systems: These systems use video deflectometers, cameras, and LiDARs to monitor bridge deformations. Computer vision techniques can be employed to process and analyze video data.

Artificial intelligence (AI) technologies may be utilized in such systems to analyze data from sensors or vision-based devices, leading to an accurate categorization of bridge health status.

### 2.1. Sensor-Based Systems

A bridge monitoring system based on Brillouin fiber-optic sensors is developed [26]. The system’s primary focus is to measure strain at multiple locations of large structures using a single optical fiber. More specifically, it utilizes distributed optical fiber sensors based on stimulated Brillouin scattering (SBS). Another optical-fiber-based technique for monitoring bridge strain is proposed [27]. The system utilizes fiber optic strain sensors developed using Fiber Bragg Grating technology to amplify the strain signal. A comparative study is conducted, comparing the results obtained from the fiber optic strain sensors with those from electrical resistance strain sensors and vibrating wire sensors. The findings indicate the superior performance of Fiber Bragg Grating-based sensors, delivering accurate readings at high scanning rates.

An IoT case study reports the successful detection of hidden defects in bridge infrastructure using tachometers, acceleration sensor technology, and fiber grating technology. The study utilized sensors to monitor critical parameters such as the deformation of bridge structure, relative displacement of arch foot level, skewback uneven settlement, main beam distortion, suspender force, ridge temperature, and strain of the main beam [28]. 

A real-time bridge monitoring system is developed using wireless technology to connect multiple bridges. The system incorporates various sensors, including load, vibration (piezo sensor, a filter, capacitor, and a bridge rectifier), water, and flex/tilt. A processing module collects data from sensors, triggering a buzzer or alarm if certain values exceed calculated threshold ranges [29]. Additionally, the authorities can access the bridge status through a mobile application developed for this purpose.

A low-cost and easy-to-implement system for measuring bridge deflection is presented in [30]. The system utilizes an inclinometer to measure the inclination of two points: the secant angle of the deflection curve and the triangle function operation. This proposed method provides high accuracy for both static and dynamic loading. Systematically, the system involves using a series of angle-measuring sensors that are placed at the edge of the bridge and parallel to the ground.

An intelligent health monitoring for a large bridge system is reported in [3]. The system uses RFID sensors for bridge identification and a set of sensors to measure various bridge health parameters, such as stress, cable force, dynamic response, fracture, and environmental indicators. The collected data are processed to assess the health status of the bridge structure. 

The use of digital twin technology to provide an accurate real-time virtual model of bridges is proposed in [31]. Those models enable the simulations of operational processes and ensure the reliability of the Structural Health Monitoring System (SHMS) in predicting bridge responses and identifying the origin of measured responses. It is worth mentioning that the SHMS focused on 12 installation points with a total number of 195 sensors. These sensors encompassed a range of devices, including accelerometers, inclinometers, and strain gauges as well as temperature, humidity, and wind sensors.

### 2.2. Vision-Based Systems

A deflection measurement system based on video deflectometer and illuminated LED targets is proposed in [32]. The system consists of a high-speed area scan monochrome camera, a fixed-focal optical lens, a laser rangefinder, an optical theodolite, and a laptop computer. 

A vision-based system developed for remote monitoring of dynamic displacements in large-scale structures, such as bridges, is developed in [33]. The system incorporates a computer connected to one or more video cameras equipped with telescopic lenses. Utilizing an object search algorithm, the system measures structural displacements by analyzing features like edges and rivets in the captured video images. A fully wireless multi-point measurement system to accurately calculate bridge displacements under live loading is introduced [34]. The system utilizes multiple synchronized wireless cameras to calculate the bridge displacements at various points. Additionally, the captured video footage is utilized for vehicle identification and tracking purposes.

A drone-based system equipped with a set of cameras is utilized for crack-displacement measurements [35]. The system employs the Three-dimensional Digital Image Correlation technique to continuously monitor the evolution of cracks.

A LiDAR-based system for estimating bridge displacement is introduced in [36]. The system utilizes point cloud data obtained through light detection and ranging scanning, which is organized into a three-dimensional space represented by interconnected nodes. Each node is linked through parent–child relationships, allowing the system to estimate the vertical displacement of the structure by locating the position of the nodes.

### 2.3. Systems Utilizing Artificial Intelligence

A cyberphysical system was proposed to monitor a highway corridor to record bridge responses [37]. This system involves three layers: physical, sensing, and internet services. It utilizes cameras and computer vision methods based on convolutional neural networks (CNN) to automate the detection and reidentification of bridge traffic and status. 

An innovative application of IoT and deep belief networks is used to detect bridges’ cracks [38]. The deep-belief networks can recognize, cluster, and generate images, video sequences, and motion-capture data, achieving a high recognition rate percentage for identifying crack types. Real-time monitoring can be achieved through a smart health monitoring technique that utilizes a fuzzy-neuro and neural network prediction hybrid architecture [39]. Vibration data are fuzzified using different linguistic terms. Based on a set of developed rules, the defuzzifier gives possible damage a digital value (yes or no). If there is a possibility, a neural network prediction algorithm is applied, which gives the level of damage value: small, medium, or large damage. Another fuzzy logic technique is proposed to calculate the crack depth and crack location by evaluating variations in the vibration constraints [40]. The fuzzy logic algorithm is applied to determine the crack location and depth of a cantilever beam. The dynamic responses of the system are utilized for damage prognoses. 

Deep convolutional neural networks can be utilized to identify damages of Reinforced Concrete (RC) members [41]. Ensemble learning algorithms are applied to structural images to estimate the structural damage and evaluate the seismic risk in earthquake-prone regions.

## 3. Proposed System Requirements

Requirements play a critical role in the system design process, serving as an important stage. There are two main types of requirements: functional and nonfunctional. Understanding and managing both functional and nonfunctional requirements lead to a well-designed system that meets expectations and standards. The proposed system must have a data acquisition unit (DAQ-Unit) and a Mobile Application (M-App).

### 3.1. System Functional Requirements

The DAQ-Unit shall be equipped with various sensors, including deflection, vibration, temperature, humidity, wind speed, and traffic count. These sensors are essential for collecting accurate bridge health parameters. The DAQ-Unit is expected to read the bridge health status as follows:

- Determining the angle of inclination concerning gravity at five equidistant points across the bridge.
- Identifying the peak vibration frequency at five equidistant points of the bridge, measured in Hertz (Hz).
- Measuring the temperature of the bridge’s surroundings in Celsius.
- Monitoring the humidity level of the bridge’s surroundings in percentage (%).
- Recording the wind speed in the bridge’s vicinity in meters per second (m/s).

Additionally, the DAQ-Unit must be capable of processing and analyzing the collected bridge health parameters effectively. The DAQ-Unit should also incorporate two cameras at each endpoint of the bridge. These cameras serve multiple purposes, including real-time bridge traffic monitoring, traffic count calculations, and providing visualization capabilities. Furthermore, the DAQ-Unit is mandated to generate report logs for readings at regular intervals—specifically, every two minutes. Additionally, it should have the ability to upload these report logs to the database for further analysis.

On the other hand, the Mobile-App shall have the ability to empower users in managing the bridge system. It shall provide clear visualizations, tables, location tracking, and live monitoring of the bridge parameters and status.

### 3.2. System Nonfunctional Requirements

The nonfunctional requirements for the bridge health monitoring and warning system encompass various aspects. The system should be extendable to function on any bridge type and length. It must also be scalable to handle hundreds of users and bridges. Furthermore, the system implementation on bridges should occur without any destruction and reconstruction. Moreover, the system should be compact and portable to ease installation and minimize operation costs. In addition, access should be restricted to authorized users. Finally, wireless data transfer capabilities must be in place for efficient communication.

**Figure 1:**
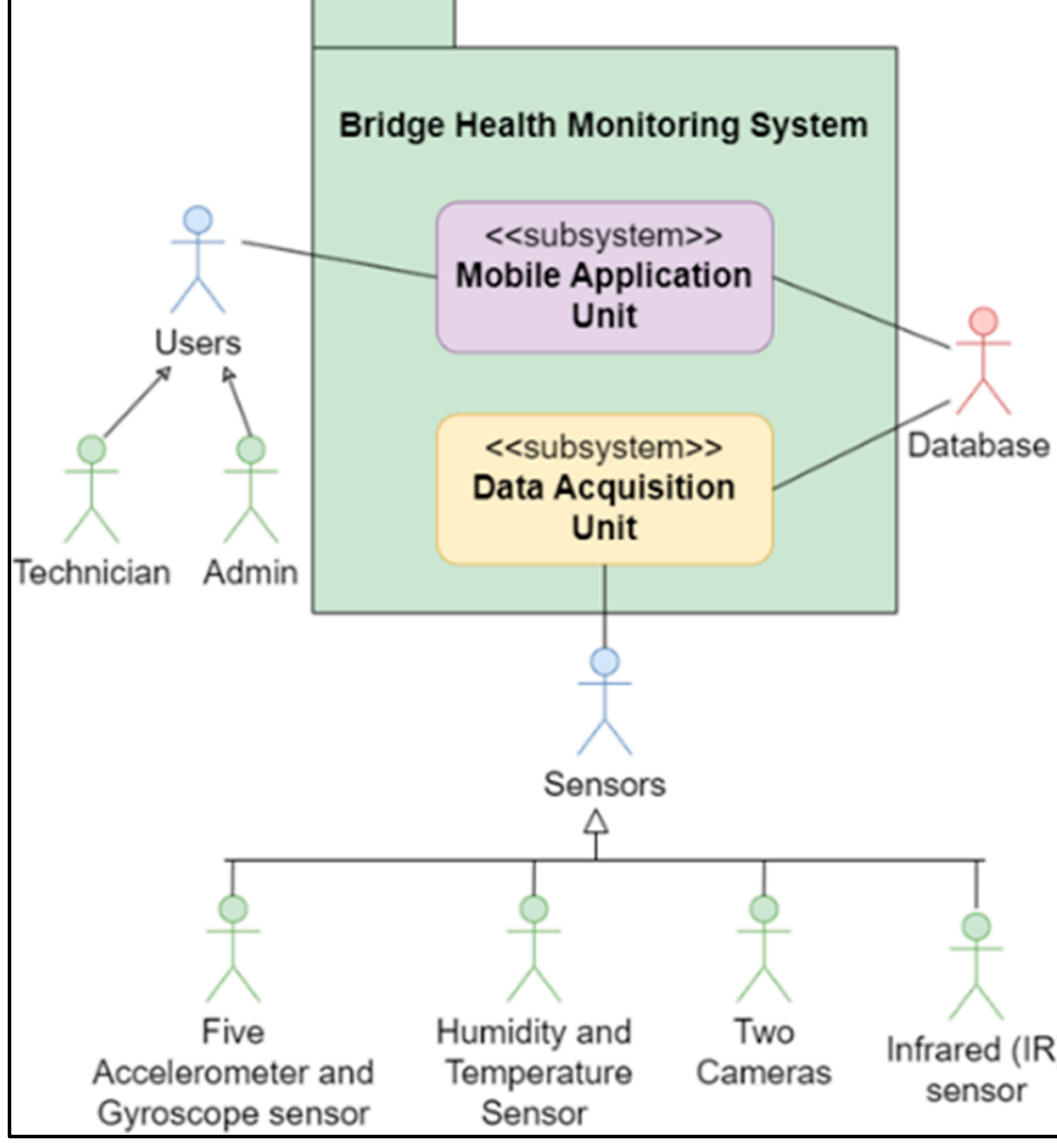
Proposed system use-case diagram.

The Bridge Health Monitoring System Use-Case Diagram shown in Figure 1 illustrates the actors, including the DAQ-Unit, Mobile-App, and Database.

## 4. Proposed System Hardware Architecture

**Figure 2:**
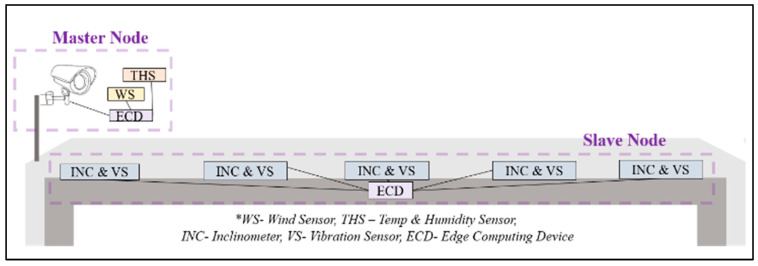
Proposed system design.

**Figure 3:**
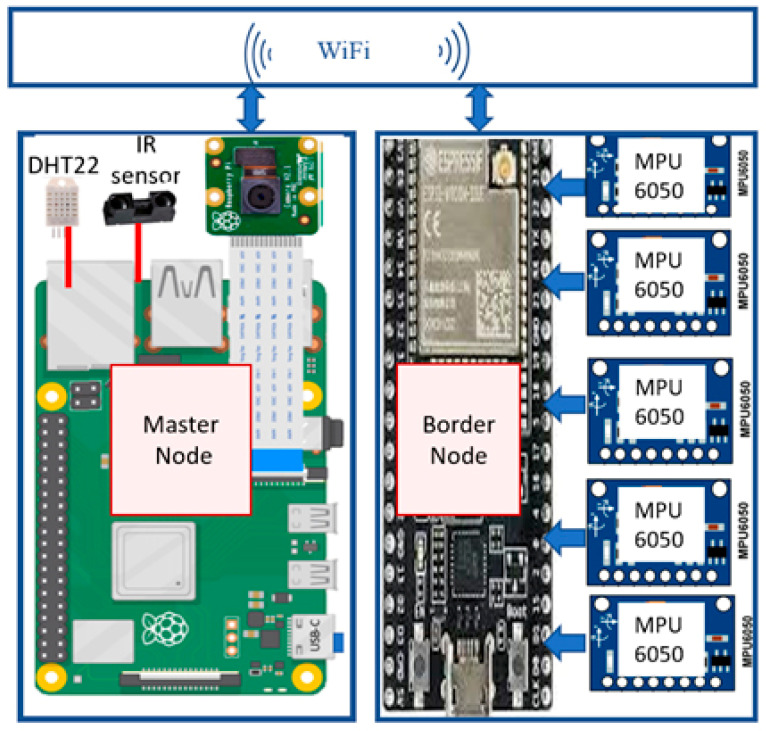
Proposed hardware architecture.

Based on the abovementioned requirements, the hardware architecture comprises two System on Chip (SoC) edge computing nodes, a Master Node and Slave Node(s), as shown in Figure 2. These nodes are equipped with a variety of sensors, including an accelerometer/gyroscope, temperature/humidity and infrared sensors, along with Raspberry Pi (RPi) Cameras and an 8x1 I2C multiplexer. The system is designed to be scalable, allowing expandability on different bridge lengths by replicating the slave node. The proposed system architecture is illustrated in Figure 3. 

The specifications and functionalities of the different components in the system are as follows:

- Edge Computing devices: Master Node consists of a Single System on Chip computing device (RPi4 model B) with a high-resolution camera and a slave node using a single chip microcontroller (ESP32). Specifications of each unit are presented in Table 1.
- Accelerometer/Gyroscope sensor (MPU6050): This sensor is used to measure acceleration and rotation in 3D space, and it incorporates an onboard digital motion processor. It can be interfaced to the edge computing device using SPI and I2C communication protocols; the latter was used in the system. The acceleration and rotation sensitivity resolutions are programmable. Five different MPU6050 sensors are placed at equidistant points of the bridge. The acceleration readings are utilized to measure the bridge’s vibration and deflection, while the gyroscope readings are used to correct the bridge’s deflection calculation. Table 1 shows the sensors’ full-scale ranges and sensitivities.
- Infrared sensor (GP2Y0A710K0F): This sensor unit is designed for distance measurement and comprises a position-sensitive detector, infrared emitting diode, and signal processing circuit. In our proposed system, the infrared sensor is integrated with an anemometer cup to create a wind sensor. The anemometer cups rotate at a speed relative to the wind speed, while the infrared sensor detects the time of each rotation, enabling the calculation of the anemometer cups’ rotational speed. Thus, the wind speed and direction can be determined. Table 1 shows the sensor’s full-scale ranges and sensitivities.
- Temperature/humidity sensor (DHT22): This sensor measures the temperature and humidity of the surrounding area at the Master Node of the bridge. Table 1 shows the sensor’s full-scale ranges and sensitivities.

## 5. Proposed System Software Architecture

The software architecture of the proposed system consists of three layers, namely, the data collection and processing layer, the data storage layer, and the application layer.

### 5.1. Data Collection and Processing Layer (DCPL)

The data collection and processing layer involves a series of tasks related to collecting readings from various sensors, processing the gathered data, and updating the data storage layer with the processed information.

#### 5.1.1. Sensors’ Readings Acquisition and System Values Computation

As previously stated, the primary objective of the system is to monitor, analyze, and classify the bridge’s health status, which relies on four key factors: surrounding temperature, wind speed, bridge deflection, and vibration. In addition, the system utilizes the camera to provide a video livestream of the bridge and to keep track of the traffic count. The temperature data will be directly collected using the DHT22 sensor, while the wind sensor, described in Section 4, will measure wind speed. The approach followed to obtain bridge deflection, vibration measurements, and traffic count measurement is described below.

#### 5.1.2. Bridge Deflection 

Keeping with the ease-of-use system requirement, deflection is a key measure in the system design. The bridge deflection can be effectively measured using the five MPU-6050 sensors, which are part of the Slave Node. These sensors continuously collect the accelerations and gyroscope data to calculate a filtered *x*-axis rotation value.

The *x*-axis rotation values (x_rotation_acc_) are computed from the accelerometer data in radians, using the x, y, and z acceleration readings, as shown in (1):

(1) $$x\_rotation_{acc}=\tan^{-1}\frac{y}{\sqrt{x^{2}+z^{2}}}$$

similarly, *x*-axis rotation values can be calculated from the gyroscope data (x_rotation_gyro_) by integrating the *x*-axis gyroscopic reading (x_gyro_), as in (2):

(2) $$x\_rotation_{gyro}=\int^{\;}x_{gyro}dt$$

both x_rotation_acc_ and x_rotation_gyro_ are then combined in the sensor fusion Equation (3) to calculate the final system *x*-axis rotation value:

(3) $$x\_rotation=0.16*x\_rotation_{gyro}+0.84*x\_rotation_{acc}$$

The complementary filter gains of the above equation are tuned to ensure accurate outcomes (zero rotation value), even under conditions of no load on the bridge.

**Figure 4:**
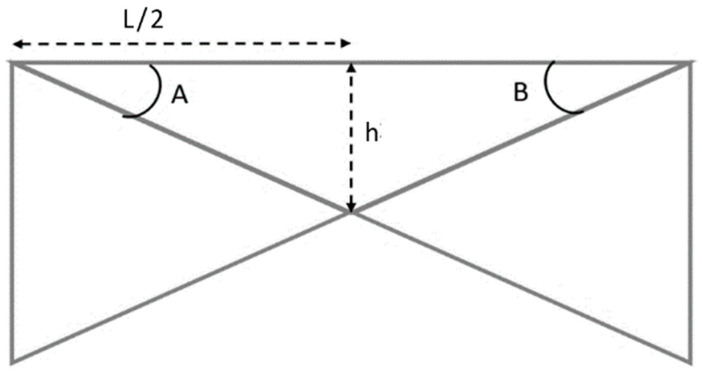
Measurement of inner deflection between two points.

Figure 4 illustrates how the internal deflection between two points (x) can be calculated using trigonometry. Given the *x*-axis rotation angles at these points (A and B), along with half of the distance between them (L/2), the deflection (h) can be calculated using (4):

(4) $$h_{\left(A,B\right)}=\frac{\sin\left(B\right)*\sin\left(90-A\right)*\frac{L}{2}}{\sin\left(A*B\right)}$$

**Figure 5:**
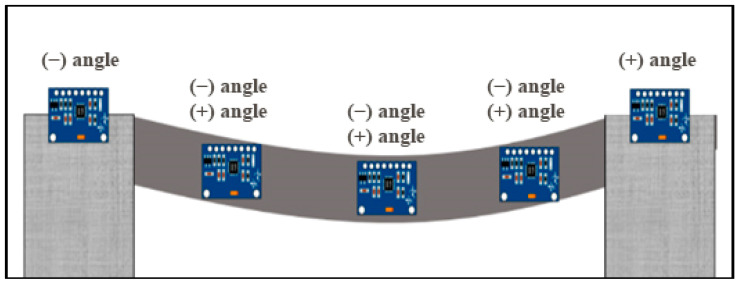
MPU5060 sensor’s locations.

Since the five MPU-6050 sensors are positioned at five equidistant points of the bridge, as shown in Figure 5, the above equation can be used to compute the deflection at different points on the bridge. Therefore, to calculate the deflection at the next sensor location, it is ideal to add the calculated deflection value at that point to the deflection value of the sensor further along the bridge. This iterative process identifies the maximum deflection of the bridge from the zero level.

**Figure 6:**
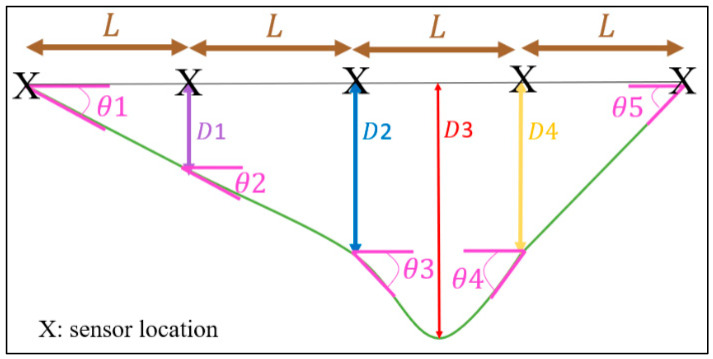
Bridge deflection values at the sensor locations (X are the sensors location, L are the distance between any two sensors).

Given that the two edge sensors always tilt in the same direction (as shown in Figure 5), the three inner sensors can tilt differently, leading to eight combinations of tilt directions and eight distinct deflection scenarios. The iterative approach used for one of these scenarios is visually represented in Figure 6. The resulting deflection values (D1, D2, D3, and D4) based on roll angles (θ1, θ2, θ3, θ4, and θ5), positions on the bridge (P1, P2, P3, and P4), and the distance between any two sensors (L) are described by Equation (5). Similar sets of deflection equations can be derived for other scenarios.

(5) $$D1=\sin\theta_{1}*L\;D2=D1+\sin\theta_{2}*L\;D3=D2+h_{\left(\theta_{3},\theta_{4}\right)}\;D4=\sin\theta_{5}*L\;P1=1*L\;P2=2*L\;P3=2*L+\frac{h_{\left(\theta_{3},\theta_{4}\right)}}{\tan\left(\theta_{2}\right)}\;P4=3*L$$

#### 5.1.3. Bridge Vibration

The vibration, on the other hand, is calculated using the MPU-6050 *z*-axis accelerometers readings. The *z*-axis is specifically chosen to mitigate unwanted noise and inaccuracies in the vibration measurements. Readings from the x and y axes may not accurately represent the actual vibration motion due to deflection and expansion. 

Fourier Transform enables decoding of the vibration data captured by accelerometers, revealing the hidden frequency patterns that provide insights into the behavior of the monitored bridge’s vibration. Initially, a set of N *z*-axis accelerometer values is sampled. 

To mitigate spectral leakage and enhance accuracy, a Hamming window is applied to the data before performing the Fast Fourier Transform (FFT). FFT is employed to convert the accelerometer’s time domain values to the vibration signal’s frequency spectrum in Hz. The major peaks of the FFT results are then extracted to indicate the crisp values for vibration at each specific point. 

The deflection and vibration values computed on the slave node(s) are then published to the master node using the Amazon Web Services (AWS) IoT Core MQTT broker.

#### 5.1.4. Traffic Count Algorithm

**Figure 7:**
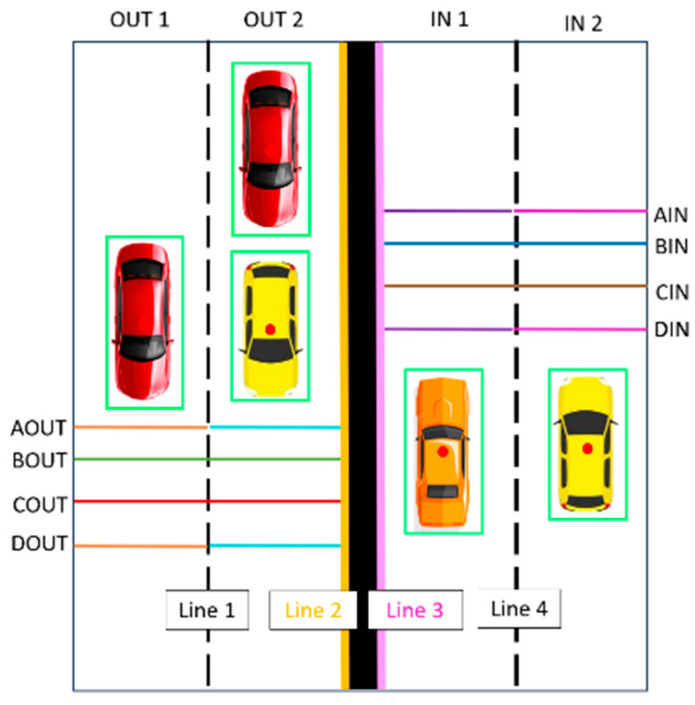
Traffic count algorithm (green boxes are the boundaries of the detected cars, the red dot is the center of each bounding box, the In and Out horizontal lines are used to make sure that each vehicle is detected only once).

A high-resolution camera module is utilized for live streaming video of the bridge and traffic flow counting. The real-time video feed enables continuous monitoring of traffic flow on the bridge, aiding in traffic management and analysis. The traffic count algorithm, which utilizes functions from the OpenCV library, uses contour detection to identify moving objects and eliminates background noise by only considering contours within a certain size range. To enhance visualization, the algorithm calculates the center of each valid contour. For accurate vehicle counting, the algorithm determines their positions relative to predefined lines on the bridge, as illustrated in Figure 7.

#### 5.1.5. Fuzzy Logic Algorithm

Fuzzy logic emerges as one of the powerful and suitable approaches for road bridge health monitoring, especially given the inherent complexity and uncertainty in such systems.

Leveraging fuzzy logic’s capacity to handle imprecise data, provide linguistic representations, and facilitate flexible decision-making, it significantly enhances the safety and reliability of road bridges. Therefore, it can contribute to a more sustainable and secure transportation infrastructure.

In this research, we developed a fuzzy logic-based algorithm to monitor the bridge health status parameters, namely, surrounding temperature, vibration, deflection, and wind speed. Fuzzy logic empowers the system to formulate rules that holistically consider these factors, enabling flexible and adaptive decision-making based on the combined effects of the inputs. Through context-aware analysis, a fuzzy-rules-engine yields interpretable results, including categories such as “Excellent”, “Safe”, “Fair”, “Critical”, or “Collapse” [24]. 

These results are easily comprehensible to engineers, maintenance personnel, and decision-makers, facilitating informed and timely actions.

**Figure 8:**
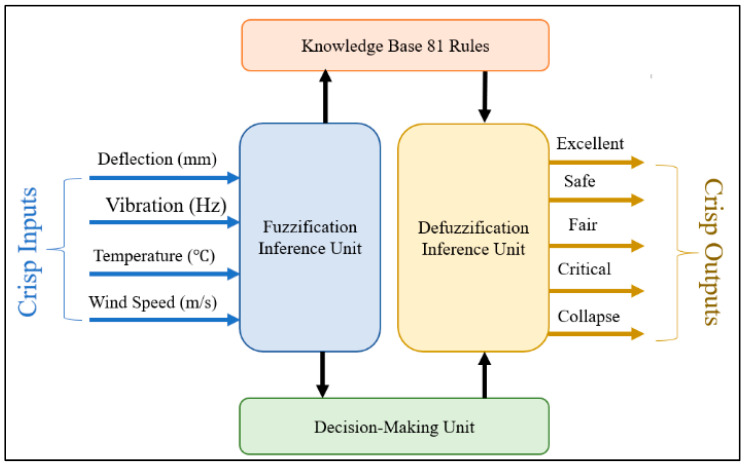
Road bridge health monitoring system fuzzy model.

Figure 8 shows the Road Bridge Health Monitoring System Fuzzy model. The following is a description of the fuzzy system for bridge health monitoring and warning: 

#### 5.1.6. Fuzzification 

The bridge’s crisp input parameters are transformed into various linguistic functions, each comprising three distinct membership functions:

- For deflection, the membership functions are “Level”, “Bending”, and “Deformed”.
- For temperature, the membership functions are “Freezing”, “Normal”, and “Very Hot”.
- For wind speed, the membership functions are “Steady”, “Gale”, and “Storm”.
- For vibration, the membership functions are “Steady”, “Movement”, and “Shaking”.

**Figure 9:**
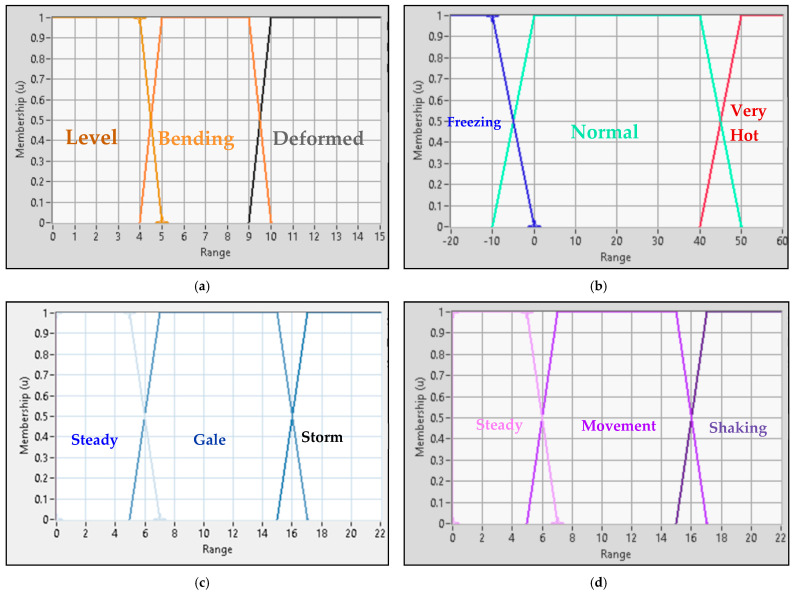
Fuzzy system crisp inputs. (**a**) Deflection. (**b**) Temperature. (**c**) Wind Speed. (**d**) Vibration.

Figure 9 shows the distribution and range of the membership functions for each crisp input parameter.

#### 5.1.7. Fuzzy Inference Rules

Utilizing inputs from the literature, field experts, and the National Bridge Inventory Code [24], a comprehensive set of rules is generated to govern the system’s decision-making process. The number of rules is a function of the number of membership functions for each input. With four inputs, and each input having three membership functions, the total number of rules amounts to 3 × 3 × 3 × 3 = 81. A sample of these rules is listed in Table 2.

#### 5.1.8. De-Fuzzification

**Figure 10:**
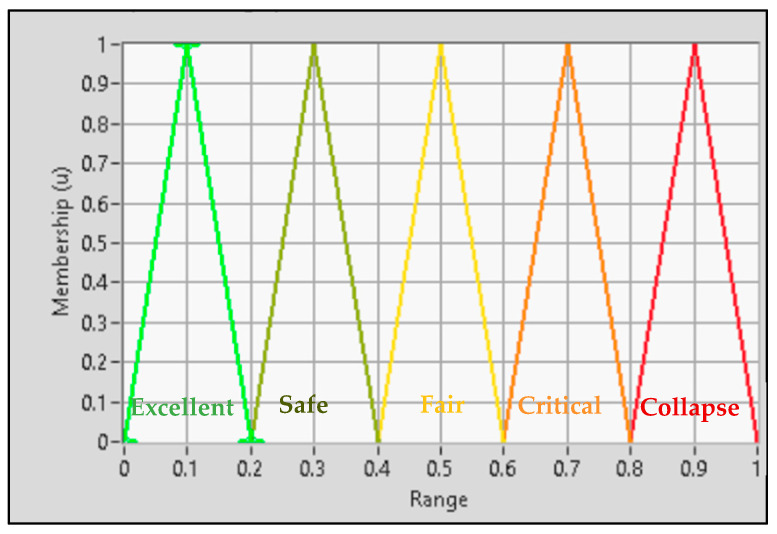
Fuzzy system crisp output.

De-fuzzification is the process of converting rule results to crisp outputs by a decision-making algorithm. The bridge state output membership functions are then categorized into five distinct levels, namely, “Excellent”, “Safe”, “Fair”, “Critical”, and “Collapse”. Figure 10 visually presents the output linguistic membership functions along with their corresponding shapes and ranges.

Deflection and vibration values that were obtained from the slave node(s) are subsequently aggregated with the measured temperature and calculated wind speed values in the master node. The master node then selects the maximum vibration and the maximum deflection. Consequently, the temperature, wind speed, maximum deflection, and maximum vibration values are fed into the fuzzy logic algorithm to determine the status of the bridge. This status and its respective health parameters are compiled into a single report log. The processed report logs are uploaded to the data storage layer. 

### 5.2. Data Collection and Processing Layer (DCPL)

The DSL utilizes Firebase, a cloud-based NoSQL database, to store the system parameters that were processed in the DCPL. The bridge database encompasses various attributes, including the bridge’s span; number of piers; count of slave nodes; and, notably, the bridge ID, which serves as a distinguishing factor among bridges. Lastly, different types of notifications are generated and stored within the notification database, based on the bridge health status level. The recipient of each notification is determined by the bridge’s specific status level.

### 5.3. Application Layer (AL)

**Figure 11:**
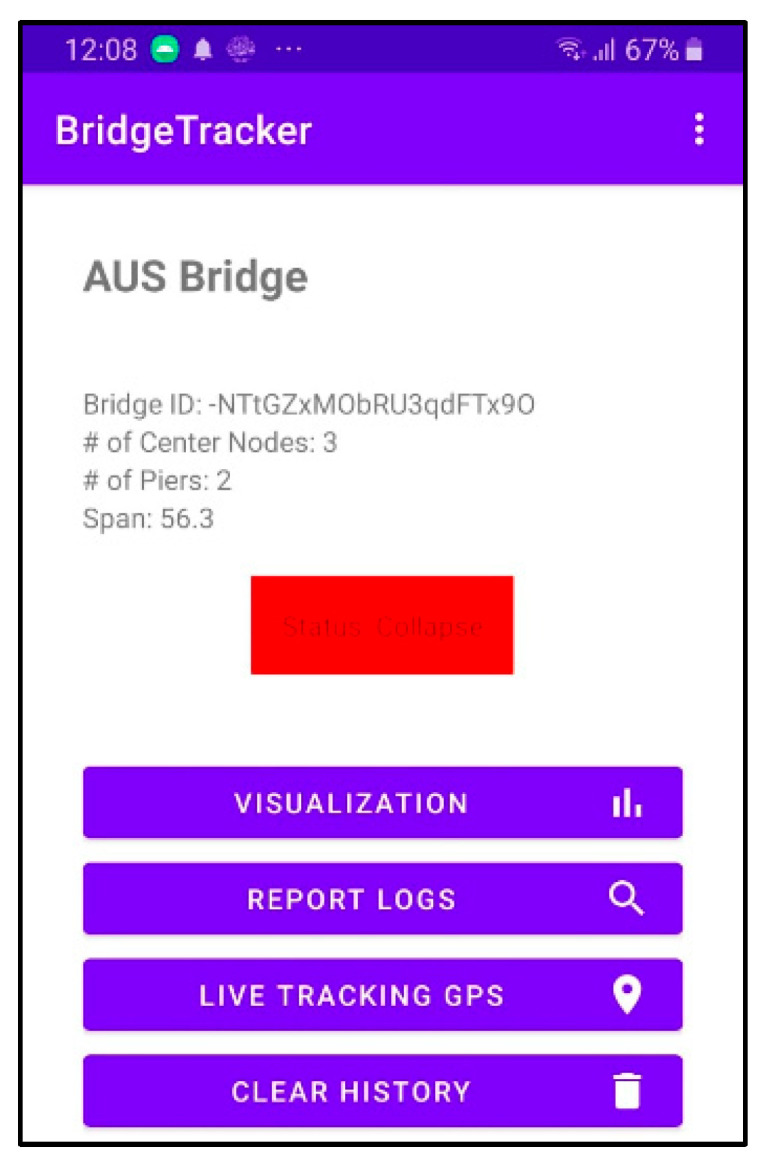
Mobile application dashboard.

This layer is responsible for delivering real-time bridge status updates to system users through a Mobile Application, accomplished by retrieving essential information from the DSL. The Mobile Application integrates with a visualization platform and Google Maps API. This application provides users with a comprehensive dashboard that contains data visualizations, detailed report logs, and the precise geographical location. The GPS live-tracking feature empowers users to monitor bridges on a map, using colored markers to indicate health status. Moreover, the application issues automated warning notifications based on the severity of bridge health status. The mobile application dashboard is illustrated in Figure 11. 

## 6. Testing, Validation, and Discussion

**Figure 12:**
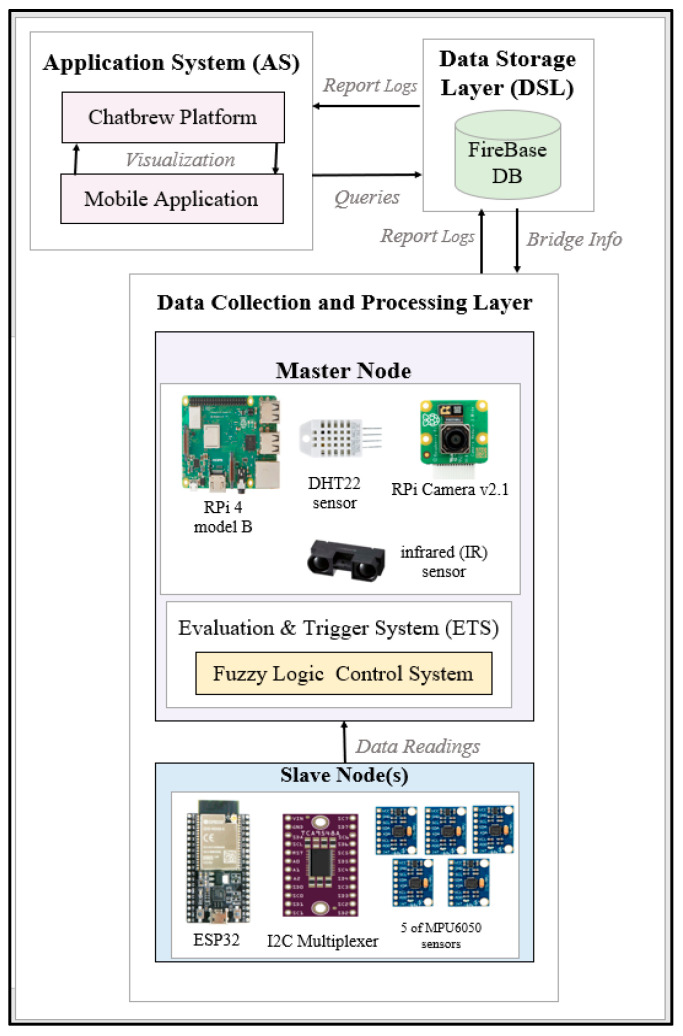
System block diagram.

The overall system’s block diagram can be seen in Figure 12. The diagram highlights the different layers in the system and their connections, as discussed in Section 5.

**Figure 13:**
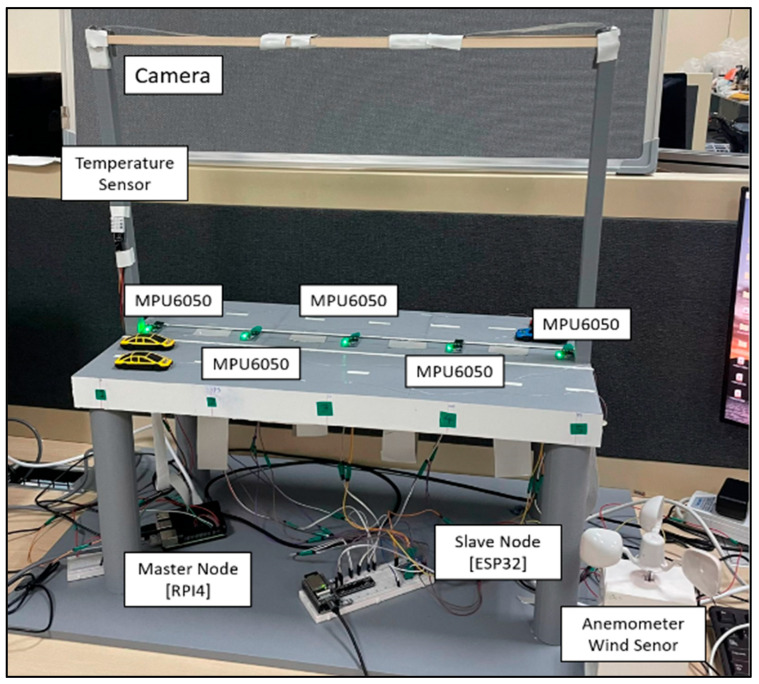
Testing bridge prototype.

To validate the system effectively, simulating various deflection, vibration, and environmental conditions on a real bridge would be challenging. Therefore, a miniature bridge prototype was constructed, designed to scale (72 cm length) to represent common real bridges. The prototype was created using AutoCAD, and printed utilizing 3D printing technology. Different 3D printing materials and thicknesses were tested to identify a suitable material capable of withstanding and supporting the weight of different miniature vehicles as well as different deflection loads for testing. All sensors and edge computing devices were attached according to the system design and programmed to replicate the full functionality of a real bridge system. All the sensors and edge computing devices were attached according to the system design and were programmed to perform the full function of a real bridge system. The bridge prototype can be seen in Figure 13.

Three different types of testing were performed for the bridge system: hardware unit testing, fuzzy logic algorithm simulation and practical testing, and overall system testing.

### 6.1. Hardware Unit Testing

In the preliminary hardware unit testing stage, we systematically examined the various inputs of the system to validate the sensors’ measurements. Evaluation factors included load variations, vibrations, traffic scenarios, and environmental conditions. Each factor was methodically examined to validate the system’s accuracy and functionality in real-world bridge health monitoring scenarios using the built prototype. 

Different loads were applied to the bridge, resulting in noticeable changes in its deflection. The prototype bridge’s actual deflection was carefully measured and compared with the deflections reported by the bridge monitoring system. The variance between these two measurements fell within the range of 1mm, emphasizing the precision and accuracy of the recorded values.

To evaluate the Fourier analysis’s capability to measure bridge vibrations, we employed vibration motors on the bridge to simulate actual vibrations. As the intensity of the applied vibrations increased, the recorded vibration values increased, validating the analysis effectiveness. Additionally, the temperature and humidity sensor values were recorded and calibrated using highly sensitive external measuring devices. Finally, the anemometer cups used to measure the wind speed were rotated at various rates, controlled by a fan with multiple speed settings. The resulting wind speed measurements accurately mirrored the adjustments in the fan speed settings. 

The effectiveness of the traffic count algorithm was verified through trials involving the passage of multiple cars across the bridge, ensuring alignment between the traffic count and the actual number of cars. Various scenarios were examined, encompassing diverse driving speeds, including both fast-moving and stationary cars. Additionally, the algorithm underwent testing under varying lighting conditions, ranging from dimly lit surroundings to well-lit environments. Furthermore, assessments covered situations with tightly packed and widely spaced cars, as well as scenarios involving identical cars.

### 6.2. Fuzzy Logic Algorithm Simulation and Testing

**Figure 14:**
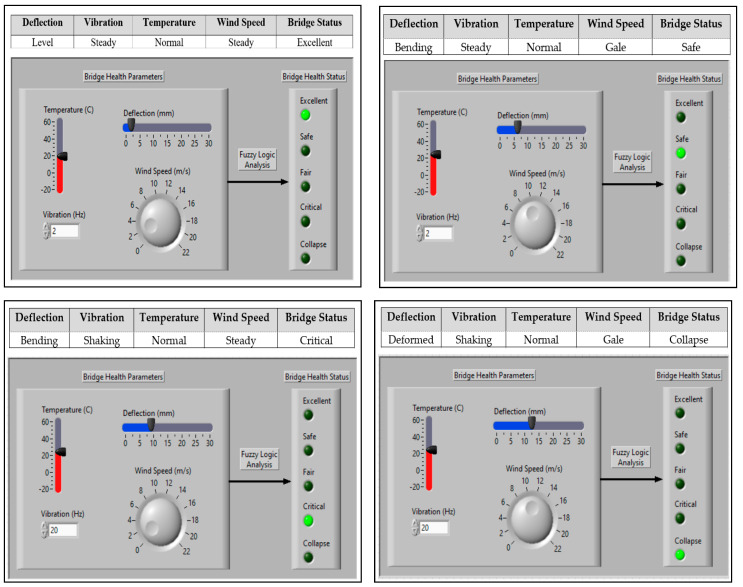
System simulation testing results.

The Fuzzy logic algorithm was implemented and tested using LabVIEW 2023 simulations. Figure 14 shows the simulation results for different rules and their respective bridge status outputs. 

### 6.3. Overall System Testing

To test the validity of the overall system, specifically its accuracy in categorizing the bridge’s health based on different loads, vibrations, and environmental conditions, the proposed fuzzy logic algorithm was developed using python libraries and loaded in the master node edge computing device.

**Figure 15:**
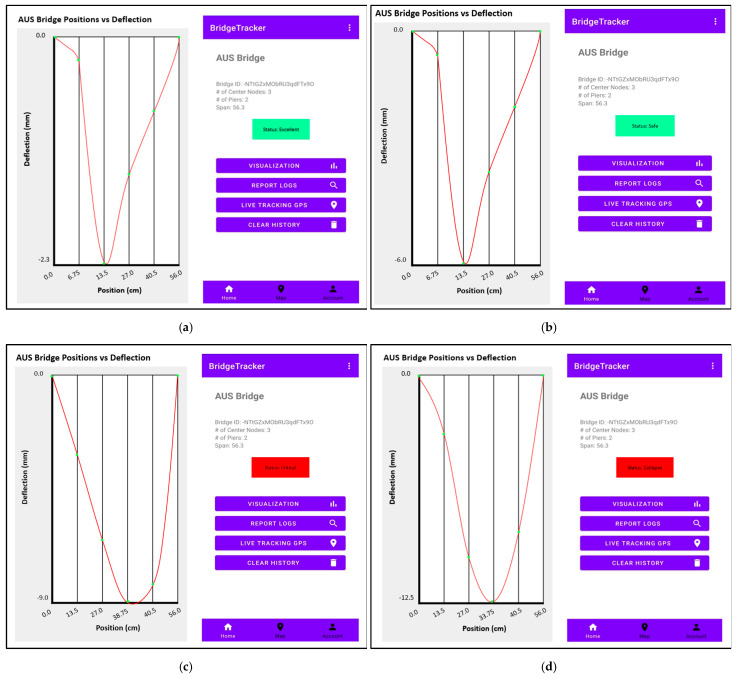
System actual testing results: (**a**) test case 1, (**b**) test case 2, (**c**) test case 3, (**d**) test case 4.

Using the same input parameter values as those utilized in the simulated four cases shown in Figure 14, the prototype underwent practical testing for bridge health status. The practical testing outcomes aligned with the simulated results. Table 3 illustrates the results of the testing for the four different cases. Using the mobile application, Figure 15 shows the actual testing results deflection graphs as well as the bridge status.

## 7. Conclusions

In this work, we propose a wireless, low-cost road bridge health status monitoring and warning system for use in rural environments and developing countries to address challenges arising from aging infrastructure and natural disasters triggered by a changing global climate. 

Leveraging advancements in sensing technology, Internet of Things (IoT), and sensor networks, this system offers an optimal blend of design parameters to address the limitations of current monitoring methods. By integrating easily relatable criteria such as deflection, vibration, temperature, and humidity with wind speed sensors alongside fuzzy logic control, the system delivers affordable, precise, and real-time assessments of bridge health. The system also provides an early warning system for rural settings where visual inspections are not easily scheduled at regular intervals. This accompanying early warning mechanism is accessible via a user-friendly mobile application and provides overseeing specialists with timely signals and indicators, enabling informed decisions and preventative actions to mitigate the potential for catastrophic bridge failures. The affordable technology system is intended to contribute to the UN Sustainable Development Goals #9—building resilient infrastructure to promote inclusive and sustainable industrialization and foster innovation, as well as #11—make cities and human settlements inclusive, safe, resilient, and sustainable. 

Furthermore, IoT technologies have the potential to streamline processes, enhance system efficiency, and elevate the overall quality of life. It is important to note that these advancements should be accompanied by carefully considering environmental impacts and the intelligent management of finite global resources. The data generated by IoT devices can support the establishment of a circular economy and offer valuable insights for decision-making among citizens and city officials. This includes proactive maintenance measures and guaranteeing transportation infrastructure’s safety and long-term viability. Incorporating IoT technology into bridge monitoring aligns with the principles of energy efficiency, circular economy, and resource optimization within the context of resilient cities. To validate our approach, our project team successfully constructed and tested a prototype of the proposed system, with the testing results aligning with our predictions. 

To address the edge computing device processing limitations, a proposed solution involves integrating a digital signal processor (DSP), enabling the utilization of more complex software algorithms like machine learning and deep learning. However, connectivity constraints typical of IoT systems, relying on Wi-Fi or GSM, pose inherent limitations, particularly in rural areas lacking such connectivity. In the context of future work, scalability can be confirmed through testing on large-scale bridges with multiple center nodes. Additionally, evaluating the system’s performance on diverse bridge structures will provide insights into its adaptability and effectiveness across various scenarios. Finally, the addition of more cameras, enabling features like crack detection, can be considered for further system improvement. 

In conclusion, IoT technologies hold promise in tackling obstacles faced by developing nations and making significant contributions toward expediting the achievement of the UN’s Sustainable Development Goals (SDGs).

## Figures and Tables

**Table 1:** Devices and specifications.

Device Type	Device Name	Specifications
Edge Devices	RPi 4 model B 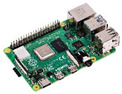	CPU: 1.5 GHz 64-bit quad core ARM Cortex-A72 processor RAM: 8 GB Networking: Wi-Fi + Bluetooth + Gigabit Ethernet port Location in the system: One at the Master Node Price per unit: USD 120
	ESP32 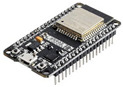	CPU: 240 MHz 32-bit dual-core LX6 microprocessor RAM: 520 KB Networking: Wi-Fi + Bluetooth Location in the system: One at the Slave Node Price per unit: USD 7
Sensors	MPU6050 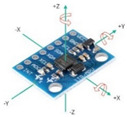	Accelerometer: Full Scale Range: ±2 g, ±4 g, ±8 g, ±16 g Sensitivity: 16,384 LSB/g, 8192 LSB/g, 4096 LSB/g, 2048 LSB/g Gyroscope: Full Scale Range: ±2500/s, ±5000/s, ±10,000/s, ±20,000/s Sensitivity: 131 LSB/°/s, 65.5 LSB/°/s, 32.8 LSB/°/s, 16.4 LSB/°/s Location in the system: Five at the Slave Node Price per unit: USD 6
	Infrared sensor (GP2Y0A710K0F) 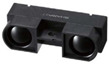	Full Scale Range: 100–550 cm Sensitivity: y = 137,500x + 1125 where y is equal to the output voltage in mV and x is equal to 1/distance in cm Location in the system: One at the Slave Node Price per unit: USD 24.5
	DHT22 digital humidity and temperature sensor 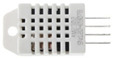	Humidity: Full Scale Range: 0~100%RH Sensitivity: 0.1%RH Temperature: Full Scale Range: −40~80 °C Sensitivity: 0.1 °C Location in the system: One at the Master Node Price per unit: USD 10
	Pi Camera 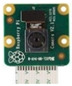	Full Scale Range: 8 Megapixel Sensitivity: 1080p x 30 Location in the system: Two at the Master Node Price per unit: USD 30

**Table 2:** Fuzzy system rules.

Deflection	Vibration	Temperature	Wind Speed	Bridge Status
Level	Steady	Normal	Steady	Excellent
Level	Steady	Very Hot	Storm	Excellent
Level	Movement	Freezing	Steady	Safe
Bending	Steady	Normal	Gale	Safe
Bending	Movement	Normal	Gale	Fair
Bending	Steady	Freezing	Storm	Fair
Bending	Movement	Freezing	Storm	Critical
Bending	Shaking	Normal	Steady	Critical
Deformed	Steady	Very Hot	Steady	Collapse
Deformed	Shaking	Normal	Steady	Collapse

**Table 3:** System testing results.

Test Case	Rule #	Deflection (mm)	Vibration (Hz)	Temperature (°C)	Wind Speed (m/s)	Bridge Health Status	
						Simulation	Actual
1	1	2.3 (Level)	2 (Steady)	24 (Normal)	2 (Steady)	Excellent	Excellent
2	4	6 (Bending)	2 (Steady)	24 (Normal)	10 (Gale)	Safe	Safe
3	6	9 (Bending)	20 (Shaking)	24 (Normal)	2 (Steady)	Critical	Critical
4	10	12.5 (Deformed)	20 (Shaking)	24 (Normal)	10 (Gale)	Collapse	Collapse

## Data Availability

Data are contained within the article.
